# Oral Health: The Need for Both Conventional Microbial and Molecular Characterization

**DOI:** 10.3390/ht6030011

**Published:** 2017-08-01

**Authors:** Elisheva Friedman, Negin Alizadeh, Zvi Loewy

**Affiliations:** 1 Department of Pharmaceutical and Biomedical Sciences, Touro College of Pharmacy, New York, NY 10027, USA; eswartz@student.touro.edu (E.F.); nalizade@student.touro.edu (N.A.); 2 Department of Microbiology and Immunology, New York Medical College, Valhalla, NY 10595, USA

**Keywords:** oral disease, systemic health, oral microbiota, microarrays

## Abstract

This study aims to consider the microbial distribution in oral disease, as well as gene analysis and expression, in elucidating: 1, the fundamental underpinnings of oral disease, and 2, the potential relationship between oral diseases and systemic health. A key focus is identifying the microbiota associated with oral disease manifestations characterized by both conventional microbiological and molecular methods. Variations in the observed microbial populations characterized by conventional and molecular approaches have been identified for caries, periodontitis, peri-implantitis, and stomatitis. The discovery of therapeutic approaches for oral disease will require comprehensive microbial and genomic analysis. This study evaluated the current state of the relevant microbial and genomic information for several prevalent oral diseases.

## 1. Introduction

Over the past 30 years, research has begun to elucidate the etiology of oral diseases. A key contributor to oral disease is the complex oral microbiome. The oral cavity is home to about 700 microbial species, many of which interact with host factors. Oral disease resulting from microbe–host interaction can cause systemic diseases, some of which may be lethal. 

The composition of the oral microbiota in dentate individuals differs significantly from that of edentulous individuals [1]. The oral microbiome of dentate individuals contains greater proportions of anaerobes and spirochetes, while the prosthetic dentures of edentate patients harbor a larger percentage of aerobic organisms, as well as yeast and lactobacilli [2,3]. This dissimilarity must be considered when examining a potential oral and systemic connection. Figure S1 summarizes the similarities and differences of microbial communities present in dentate and edentulous populations [1].

The study of genomes, both human and microbial, can provide insight into the etiology of disease, as well as help elucidate potential approaches for therapeutic intervention. DNA microarrays, also referred to as gene chips, allow for massively parallel, rapid screening of thousands of genes. The methodology allows for the identification of genes that are expressed differentially. Gene chips show the modulation of mRNA expression levels, suppression of gene expression, and activation of gene expression. This technology allows for large-scale genomic analysis between patients with and without a given disease.

The objectives of this paper include: (i) to provide a perspective for the role of microbes in several oral diseases; (ii) to identify differences between microbes detected by classical microbiological approaches as compared to molecular analyses; and (iii) to provide a genomic perspective on the relationship between oral and systemic health.

## 2. Caries

Dental caries is one of the most widespread chronic diseases [4]. Current epidemiological studies indicate a marked increase in the prevalence of dental decay among all age groups [5]. Dental caries is a polymicrobial infection that results from an imbalance of the dynamic metabolic process in the dental biofilm [6]. The ultimate harm to the teeth is not apparent until the mineralization balance in the supragingival biofilm is disturbed, affecting homeostasis in the biofilm [7]. Classical experimental techniques have provided significant information on the microorganisms associated with dental caries. Tooth decay is initially activated by early colonizers such as *Streptococcus oralis,* followed by adherence of *Streptococcus sobrinus* and *Stretococcus mutans* [8]. 

More recently, the Human Oral Microbe Identification Microarray (HOMIM) has been used to provide a more comprehensive description of the biofilm composition associated with the oral cavity. It can detect pathogens regardless of whether they can be cultivated or not [6,8]. This new metagenomic approach may also be useful to assess the dynamic process of the metabolic activity of biofilms. Microarrays hold high promise for advancements in oral biology. They are specifically useful for the diagnosis, prevention, and monitoring of microorganisms in the oral cavity, which should lead to better management of patients’ oral health [9].

One of the areas in clinical dentistry where microarrays have proved to be very effective is the analysis of the oral microbiota in pediatric patients between the age of three months and three years [10]. Using gene chip analysis, the relationship between the microorganisms and the presence or absence of caries can be assessed. Microarray analysis has shown that many of the bacteria that colonize the oral cavity at three months of age continue to be present at three years of age. A few of the early bacteria cease to exist as the baby matures, and correspondingly, new pathogens start colonizing at age three for more than 50% of children [10]. Although the microbiota composition at three months of age is unrelated to caries development at a later age, several pathogens present in the oral biofilms of three-year-olds can be linked to caries [10].

DNA sequencing has also been used to examine the bacterial community associated with caries. The 454 sequencing technology (454 Life Sciences, Branford, CT, USA) was used to evaluate microbial diversity influenced by the pH of cavitated lesions [11]. pH within a dentinal cavitated lesion was found to significantly affect the microbial population of 42% of the caries-associated bacteria.

Interestingly, bacterial culture methodologies continue to provide information on the bacterial composition associated with caries. An anaerobic culture of bacteria associated with caries has been very valuable in elucidating the bacterial population. Using rich non-selective media and anaerobic incubation has resulted in the improved detection of Actinobacteria as compared to PCR and cloning/sequencing analysis [12]. Table 1 summarizes the representative bacteria associated with dental caries. 

## 3. Salivary Diagnostics

Saliva is a biological fluid secreted by the salivary glands. Saliva contains bacteria originating from the surfaces of various intraoral surfaces, including teeth, gingival crevices, tongue and buccal mucosa [13]. Bacterial species prevalent in saliva are summarized in Table 2.

Saliva is also a rich source of proteins, mRNA, miRNA (non-coding RNA), and antibodies [14,15,16,17,18,19]. It therefore has immense diagnostic potential, both for identifying individuals with a given disease and for tracking patients’ disease progression and treatment response. An obvious advantage of this strategy is that the collection of saliva is a non-invasive procedure. 

Microarrays have proven to be excellent experimental platforms to analyze saliva [14,17]. The results provided by microarrays are rapid and remarkably precise, two factors that are key in diagnostics. Salivary analysis by microarrays has been used to identify and track numerous systemic diseases. For example, fiber microarrays have been used to demonstrate the altered salivary protein profile of patients with asthma and cystic fibrosis [20], whereas a microsphere-based array could not identify a significant alteration. Investigators have even developed an effective discriminatory salivary test, in which microarray detection of the downregulation of five salivary mRNA biomarkers reliably indicates the presence of ovarian cancer [21].

Many other diseases have been shown to impact the saliva’s contents. While microarrays have not yet been used as a diagnostic tool for these clinical states, there is certainly potential for the development of such procedures. There are numerous diseases whose impact has been illustrated: 19 genes are differentially expressed in the saliva of Sjogren’s syndrome patients [22], and autoantigens in the saliva differ according to the subtype of systemic lupus erythematous [23]. The blood-borne pathogens, human immunodeficiency virus [24] and hepatitis C virus antibodies can be found in salivary concentrations that correlate to the systemic viral load [25]. With respect to metabolic diseases, 65 proteins are differentially expressed in the saliva of patients with type II diabetes mellitus [26]. There is potential for advancement in cancer salivary diagnostics as well; proteins, mRNA and miRNA have been shown to have distinct patterns in patients with breast cancer [27], head and neck squamous cell carcinoma [28], lung cancer [29], oral squamous cell carcinoma [30], and resectable pancreatic cancer [31] (Table 3).

## 4. Gingivitis and Periodontitis

The composition of the oral microbiota has been investigated for well over half a century. With the advent of molecular diagnostic assays including DNA probes and PCR, as well as immunoassays designed to characterize the association between the subgingival microbiota and the levels of biomarkers released by tissues and cells measured in gingival crevicular fluid (GCF), significant progress has been made in elucidating the composition of the subgingival microbiota. The role of five main microbial complexes in the gingival biofilm was characterized and described using a checkerboard DNA–DNA hybridization [32]. Using cloning and Sanger sequencing, as well as next-generation sequencing techniques, suggested that cultivatable as well as not-yet-cultivatable microbial species are involved in the etiology of periodontitis [33,34]. Based upon current information, it appears that periodontal disease is the result of infection, with a relatively small number of interacting species. Periodontal microbiota identified by classical microbiology methods as well as molecular approaches are summarized in Table 4. A systemic review was reported that showed the association of 17 species/phylotypes from the Bacteria domain, the *Candidatus Saccharibacteria* phylum, and the Archaea domain with the etiology of periodontitis [35].

In clinical practice, microarrays can be used to detect and quantify the specific pathogens responsible for periodontitis [37]. They can also be utilized to identify whether the pathogens are the ones more likely to be associated with refractory periodontitis, and to assess the efficacy of periodontal therapy [38]. Kinney et al. [39] demonstrated that by examining the salivary concentrations of pathogens, matrix metalloproteinase-8 and -9 (MMP-8, MMP-9), calprotectin, osteoprotegerin (OPG), tumor necrosis factor (TNFα), interferon (IFN), and numerous interleukins (ILs), one can examine the progression or non-progression of periodontal disease. Furthermore, a number of these salivary solutes can be used predictively: high concentrations of pathogens *Fusobacterium nucleatum*, *Campylobacter rectus* and *Prevotella intermedia* predict disease progression, while low levels of MMP-8, MMP-9, OPG and IL-1β predict stability [39].

From a research perspective, microarrays are valuable tools for gaining further understanding of the pathophysiology of periodontitis. For example, they have been used to demonstrate the involvement of long non-coding RNAs (IncRNAs) in the pathogenesis, with the upregulation of 4313 and downregulation of 4612 lncRNAs in chronic periodontitis tissue [40], and to establish that both chronic and aggressive periodontitis have similar gene expression profiles, with limited differences in the gingival transcription patterns [41]. Interestingly, Schaefer [42] used microarray analysis to reveal that certain genetic variations thought to bear connection to periodontitis do not, in fact, predispose patients to development of this disease [42]. Microarrays have even been used to identify an apoptotic pathway as a potential anti-periodontitis pharmacological target [43].

Microarrays are often used in comparative studies to determine altered levels of bacterial pathogens, or the expression of genes in patients with and without periodontitis. Belstrom et al. demonstrated that certain bacterial taxa are upregulated only in the saliva of periodontitis patients, rather than the saliva of patients with good oral health [44]. MicroRNA (miRNA) has been a large focus in this subfield; Xie discovered that in comparison to healthy gingival tissue, inflamed gingival tissue caused the upregulation of 91 miRNAs and downregulation of 34 miRNAs, all over two-fold [45]. Similarly, Lee showed that six miRNAs are upregulated in periodontitis [46]. It has been suggested that salivary miRNAs will comprise the next generation of diagnostic periodontitis tests, but it will be necessary to first develop standardized tests and protocols [47].

## 5. Peri-Implantitis

Reconstructive dentistry today is largely facilitated by dental implants, as compared to fixed or removable partial dentures. During the past decade, the rate of growth for osseointegrated dental implants in Americans has been estimated at approximately 500,000 per year [48]. The increase in the number of implants has also given rise to a corresponding increase in clinical problems associated with the implants. Two diseases, peri-implant mucositis and peri-implantitis, have emerged as diseases associated with dental implants. Peri-implant mucositis is similar to gingivitis; it is characterized by inflammation of the mucosa without a corresponding bone loss. Peri-implantitis is characterized as a more severe inflammatory lesion, with a loss of supporting bone around an implant. Like gingivitis and periodontitis, peri-mucositis and peri-implantitis are also initiated with microbial infections. Peri-implantitis can result in a loss of the implant as well as infection of other implants or the remaining natural teeth. Peri-implantitis is asymptomatic, since pain is infrequent. As a consequence, patients do not recognize that a problem exists until the onset of implant mobility. Of all implant recipients, the prevalence of peri-implantitis has been estimated at 28–56% [48].

Peri-implantitis is a polymicrobial infection [48]. There are relatively few studies that have used molecular approaches to characterize the peri-mucositis and peri-implantitis microbiota [49]. The use of 16S-based sequencing suggests that the peri-implant microbiome may be distinct from that of the periodontal microbiome. To date, no deep metagenomic sequencing analyses of peri-implantitis samples have been reported. Table 5 summarizes the microbiota associated with peri-implantitis, as identified by both conventional as well as molecular methodologies [50,51].

Limited studies focused on the genomic and gene expression profiles for peri-implantitis have been reported [52]. Peri-implant healing has been associated with the differential expression of several genes, including cytokines, growth factors, transcription factors and secretory products.

## 6. Stomatitis

In denture stomatitis (DS), the denture is a major reservoir of many microbes, especially *Candida albicans*, a chronic source of infection. The denture surface provides a matrix that allows for the development of a pathogenic yeast biofilm. The surface under a denture is more acidic and less open to the oral mucosal saliva. This provides for an ideal environment for *C. albicans* enzymatic activity, which leads to *C. albicans* biofilm colonization and resistance. *C albicans* biofilms are the reservoirs for infection; they are enclosed within their own extracellular matrix (ECM) and attached to the surface. 

The biofilm composition and structure protects the fungi from the environment, physical and chemical stress agents, and provides resistance to antifungal agents as well. Indeed, the biofilm is up to 1000-fold more resistant to antifungal agents than planktonic free-floating cells [53]. Planktonic cells are thin, and have to undergo irreversible genetic changes to provide resistance. However, biofilms are 200–300 nm thick, and are able to persist due to their physical presence in a phase-specific manner, regardless of genetic alteration. The biofilm resistance is correlated with efflux pumps that develop during the intermediate phases of biofilm formation and extracellular matrix (ECM) production.

Table 6 lists the microbes associated with stomatitis that have been identified by conventional microbial methodologies. The microbes related to stomatitis that have been identified by molecular analytical approaches are catalogued in Figure S1. The contribution of molecular biology to the elucidation of stomatitis-related microbes is readily apparent upon comparing the microbial population identified by conventional methods with those characterized by molecular approaches.

To better understand the molecular underpinnings of DS, microarray technologies have been used to assess gene expression variability, from an early stage of biofilm growth to the maturity associated with DS. The genomic composition of the mucosa of healthy denture wearers has been compared to the mucosa of denture wearers with DS, and it was found that more than 3000 genes are subjected to transcriptional expression changes in the diseased state as compared to the healthy state [55]. Among those genes differentially expressed, 71 genes were downregulated. These genes code for neutrophil, lymphocyte, monocyte, keratins, and epithelial adhesion molecules, all of which mediate an innate response and the release of inflammatory mediators in DS. In contrast, 235 genes were upregulated in response to hyphae that were inserted by *Candida albicans* biofilm into underlying epithelial layers. 

All of the upregulated genes increase the ability of *C. albicans* to bind and penetrate the oral epithelial mucosa, and so lead to the increase in inflammatory response. Knowing what genes are expressed differently, and whether they are upregulated or downregulated, and also knowing at what phase of biofilm formation these changes of gene expression are developed, provides an understanding as to why biofilms are mostly antifungal resistant.

## 7. Conclusions

A systems approach has been presented to catalogue the microbes involved in several oral diseases. Both conventional microbiology as well as new molecular analytical methodologies are needed to comprehensively define the representative microbial populations in oral disease. Discovery of the right therapeutic interventions will require microbiology classification, DNA information, clinical information (medical records) and lifestyle information. The hope is that analyzing the microbiota, the microbial genomes, and the host human genome, alongside performing functional genomic analysis, will reveal critical pathways associated with the initiation and progression of oral disease, and provide candidate targets for drug therapies.

## Figures and Tables

**Table 1:** Microbiota associated with caries.

Conventional Methods	Molecular Methods	References
*Streptococcus* *Lactobacillus* *Actinomyces*	*Streptococcus* *Lactobacillus* *Actinomyces* *Bifidobacterium* *Propionibacterium* *Veillonella* *Selenomonas* *Atopobium*	[11,12]

**Table 2:** Microbiota prevalent in saliva.

Bacteria	References
*Streptococcus*	[13]
*Granulicatella*	
*Neisseria*	
*Rothia*	
*Prevotella*	

**Table 3:** Cancer salivary biomarkers.

Cancer	Up-Regulation	Down-Regulation	Reference
Breast Cancer	Vascular Endothelial Growth Factor (VEGF) Epidermal Growth Factor (EGF) Carcinoembryonic Antigen (CEA)		[27]
Head and Neck Squamous Cell Carcinoma	miRNA-9 miRNA-191	miRNA-134	[28]
Lung Cancer	CCNI FGF19 FRS2 GREB1 EGFR		[29]
Oral Squamous Cell Carcinoma	miRNA-24 miRNA-27b	miRNA-136 miRNA-147 miRNA-1250 miRNA-148a miRNA-632 miRNA-646 miRNA668 miRNA-877 miRNA-503 miRNA-220a miRNA-323-5p	[30]
Resectable Pancreatic Cancer	miRNA-940	miRNA-3679-5p	[31]

CCNI: Cyclin I; FGF19: Fibroblast Growth Factor 19; FRS2: Fibroblast Growth Factor Receptor Substrate 2; GREB1: Growth Regulation by Estrogen in Breast Cancer 1.

**Table 4:** Microbiota associated with periodontitis.

Conventional Methods	Molecular Methods	References
*Porphyromonas*	*Bacteroides*	[35,36]
*Prevotella*	*Firmicutes*	
*Tannerella*	*Proteobacteria*	
*Treponema*	*Spirochaeta*	
*Fusobacterium*	*Candida*	
*Campylobacter*		

**Table 5:** Microbiota associated with peri-implantitis.

Conventional Methods	Molecular Methods	References
*Bacillus* *Aggregatibacter* *Candida* *Staphylococcus*	*Enterococcus* *Streptococcus* *Porphyromonas* *Fusobacterium* *Prevotella* *Bacillus* *Neisseria* *Kingella* *Veillonella* *Capnocytophaga* *Paracoccus* *Leptotrichia* *Tannerella* *Treponema*	[49,50,51]

**Table 6:** Microbiota associated with stomatitis.

Conventional Methods	References
*Candida*	[54]
*Staphylococcus*	
*Enterobacter*	
*Pseudomonas*	

## Supplementary Materials

The following are available online at www.mdpi.com/2571-5135/6/3/11/s1, Figure S1: Microbial flora present in dentate and edentulous populations.
