# Supplementary material for: Oral Health: The Need for Both Conventional Microbial and Molecular Characterization
Source: High Throughput. 2017 Aug 1;6(3):11. doi: 10.3390/ht6030011 (PMC5734190; doi:10.3390/ht6030011)
Supplement: Supplementary file 1 [file high-throughput-06-00011-s001.pdf]

Review

Oral and Systemic Health: A Microbial and Genomic Perspective

Elisheva Friedman [1], Negin Alizadeh [2] and Zvi Loewy [3,4*]

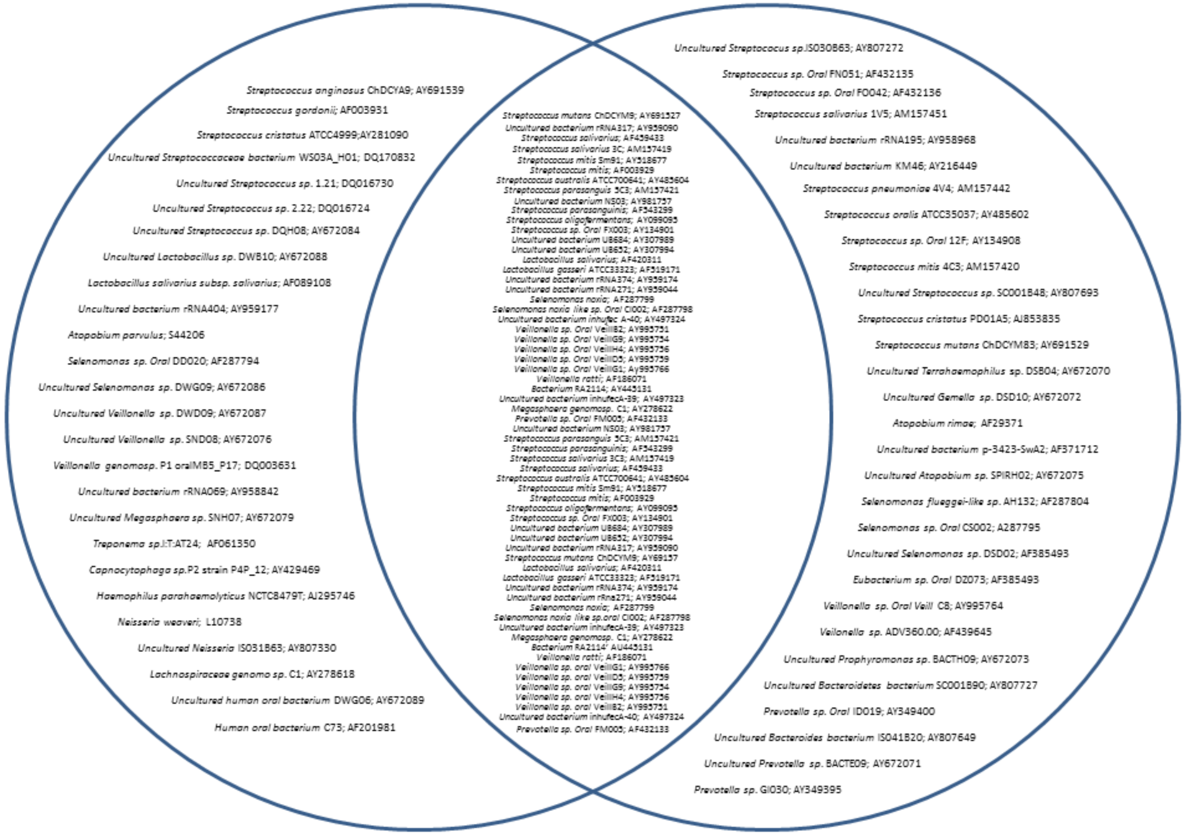

Figure S1: Microbial flora present in dentate and edentulous populations.
